# The involvement of miRNAs in the enhanced immune response of oysters via haemocyte-mediated immune priming

**DOI:** 10.3389/fimmu.2026.1753252

**Published:** 2026-03-04

**Authors:** Xiaoxu Zhou, Lixin Guo, Weilin Wang, Lingyuan Song, Yuefeng Dai, Jiajun Zuo, Lingling Wang, Linsheng Song

**Affiliations:** 1 Liaoning Key Laboratory of Marine Animal Immunology and Disease Control, Dalian Ocean University, Dalian, China; 2 Liaoning Key Laboratory of Marine Animal Immunology, Dalian Ocean University, Dalian, China; 3 Dalian Key Laboratory of Aquatic Animal Disease Prevention and Control, Dalian Ocean University, Dalian, China; 4 Laboratory for Marine Fisheries Science and Food Production Processes, Qingdao Marine Science and Technology Center, Qingdao, China

**Keywords:** Cgi-miR-1175-P6/P7-y, Crassostrea gigas, haemocyte proliferation, immune priming, miRNA

## Abstract

Immune priming enhances innate immunity, leading to a sustained and augmented response upon secondary challenge. The emerging evidence has highlighted the crucial role of endogenous microRNAs in trained immunity of vertebrates. However, the regulatory role of miRNAs in immune priming of invertebrates remains largely unknown. In the present study, the miRNA expression profile in the haemocyte-mediated immune priming of oysters *Crassostrea gigas* was examined. There were 115 up- and 212 down-regulated miRNAs screened after primary stimulation, and 107 up- and 103 down-regulated miRNAs identified after secondary stimulation. Among these, 67 miRNAs were differentially expressed in both the primary and secondary stimulations of *Vibrio* sp*lendidus*. Putative immune enhancing miRNAs (*Cgi*-miR-1175-P6/P7-y and novel-0095-3p) showed lower expression upon secondary stimulation compared to the primary response. KEGG analysis indicated that target genes of *Cgi*-miR-1175-P6/P7-y and novel-0095-3p were enriched in cell proliferation-related pathways and metabolic pathways. Target prediction suggests that *Cgi*-miR-1175-P6/P7-y and novel-m0095-3p may target genes involved in cell survival (*Cg*TEP, *Cg*IAP), cell proliferation (*Cg*CDK6 and *Cg*CDK14) and pattern recognition (*Cg*SCARF2), respectively. Through *in vivo* injections of *Cgi*-miR-1175-P6/P7-y mimics, both the rate of EdU^+^ haemocytes and the mRNA expression levels of its target genes (*Cg*CDK6, *Cg*CDK14 and *Cg*SCARF2) were significantly reduced in mimics-treated group after *Vibrio* sp*lendidus* stimulation, whereas the opposite effects were observed in the *Cgi*-miR-1175-P6/P7-y inhibitor-treated group. These findings highlight the regulatory role of miRNAs in immune priming and identify *Cgi*-miR-1175-P6/P7-y as a key post-transcriptional regulator of haemocyte proliferation.

## 1 Introduction

The oyster (*Crassostrea gigas*) is a dominant aquaculture species in China (1). However, disease outbreaks caused by pathogenic infections severely hinder the development of the oyster aquaculture industry, with the lack of effective disease control measures. As one of the major bacterial pathogens for oysters, *Vibrio* sp*lendidus* has been shown to cause mortality rate of 65–85% (2) and significantly increase haemocyte mortality (3). Previous studies have revealed that the innate immune system of *Crassostrea gigas* has evolved diverse mechanisms to launch an enhanced defense response upon recurrent pathogen encounters (4), which is known as immune priming in invertebrates (5–7) and trained immunity in vertebrates (8). Although research on immune priming in mollusks has advanced considerably (9–12), the underlying molecular mechanisms and temporal immune dynamics remain highly divergent. For instance, whereas immune stimulants such as poly(I:C) induce a sustained intracellular antiviral state in oysters (13), inactivated bacteria trigger a transient immune defense that rapidly subsides after pathogen clearance. The regulatory mechanisms of immune priming in oyster have attracted growing attention, which holding promise for the development of disease control strategies.

Emerging evidence has demonstrated that epigenetic reprogramming, notably mediated by microRNA (miRNA), plays a critical role in establishing innate immune memory (14, 15). As essential post-transcriptional regulators, miRNAs orchestrate immune responses by dynamically targeting genes to regulate both the immune initiation and immune effect. During the initiation and maintenance stage of immune response, miRNAs coordinate cellular proliferation and metabolic reprogramming to establish the cellular foundation and energetic support required for effective immunity. In humans, miR-32-5p promotes breast cancer cell proliferation by upregulating c-MYC (16). Meanwhile, β-glucan-induced trained immunity requires miR-9-5p-dependent suppression of IDH3α, thereby inhibiting KDM5 to drive integrated metabolic-epigenetic reprogramming (17). In the effector phase of immune response, miRNAs directly regulate immune cell functions such as cytokine production and phagocytosis. Chronic lipopolysaccharide stimulation induces miR-222 up-regulation, which suppresses a key subunit of chromatin remodeling complexes and effectively silences sustained inflammatory responses, thereby establishing macrophage tolerance (14). miR-133 has been documented to enhance phagocytic activity in *Apostichopus japonicus* by specifically targeting *Aj*IRAK-1 (18). Collectively, these findings demonstrate that miRNAs play a pivotal role in regulating innate immune memory by integrating metabolic reprogramming, epigenetic remodeling, and effector functions across all phases of the immune response.

Increased cellular proliferation is a characteristic of immune priming across most invertebrates (19). After priming with pre-inactivated *V.* sp*lendidus*, a substantial increase in both total haemocyte count (THC) and newly generated circulating haemocytes was detected in *C. gigas* at 6 h after secondary stimulation (12). Meanwhile, primed *Biomphalaria snails* also exhibited a pronounced increase in THC at 24 h after secondary challenge (10), whereas primed white shrimp (*Litopenaeus vannamei*) showed a markedly higher proliferative cell ratio and mitotic index in hematopoietic tissues at 3 d post re-exposure to *Vibrio alginolyticus* (6). While the role of post-translational modifications (e.g., H3K4me3) in the innate immune memory of oysters during immune priming is recognized (20), the role of post-transcriptional regulation mechanisms remains elusive. Given the critical role of miRNAs in epigenetic reprogramming during trained immunity, the regulatory role of miRNAs in immune-activated cells may represent an indispensable component for elucidating the mechanisms underlying enhanced immune response in oysters. The objectives of this study were (1) to explore the alteration of miRNAs after the *V.* sp*lendidus* stimulation (2), to identify putative immune enhancing and the immune memory-related differentially expressed (DE) miRNA (3), to uncover the role of key miRNAs in regulating haemocyte proliferation during immune priming in oysters.

## 2 Materials and methods

### 2.1 Animal treatments

Two-year-old oysters were acquired from an aquaculture farm in Dalian and acclimatized for one week in the laboratory. Oysters were cultured at 15-20 °C in aerated seawater filtered through a 300−mesh nylon sieve. A 50% daily water exchange was performed, and oysters were fed with spirulina powder thoroughly dissolved in seawater. The *V.* sp*lendidus* strain maintained in our laboratory was cultured by inoculating into 2216E liquid medium. All procedures followed ethical guidelines approved by the Dalian Ocean University Ethics Committee.

### 2.2 Immune stimulation of oysters and haemocytes collection

**Figure 1:**
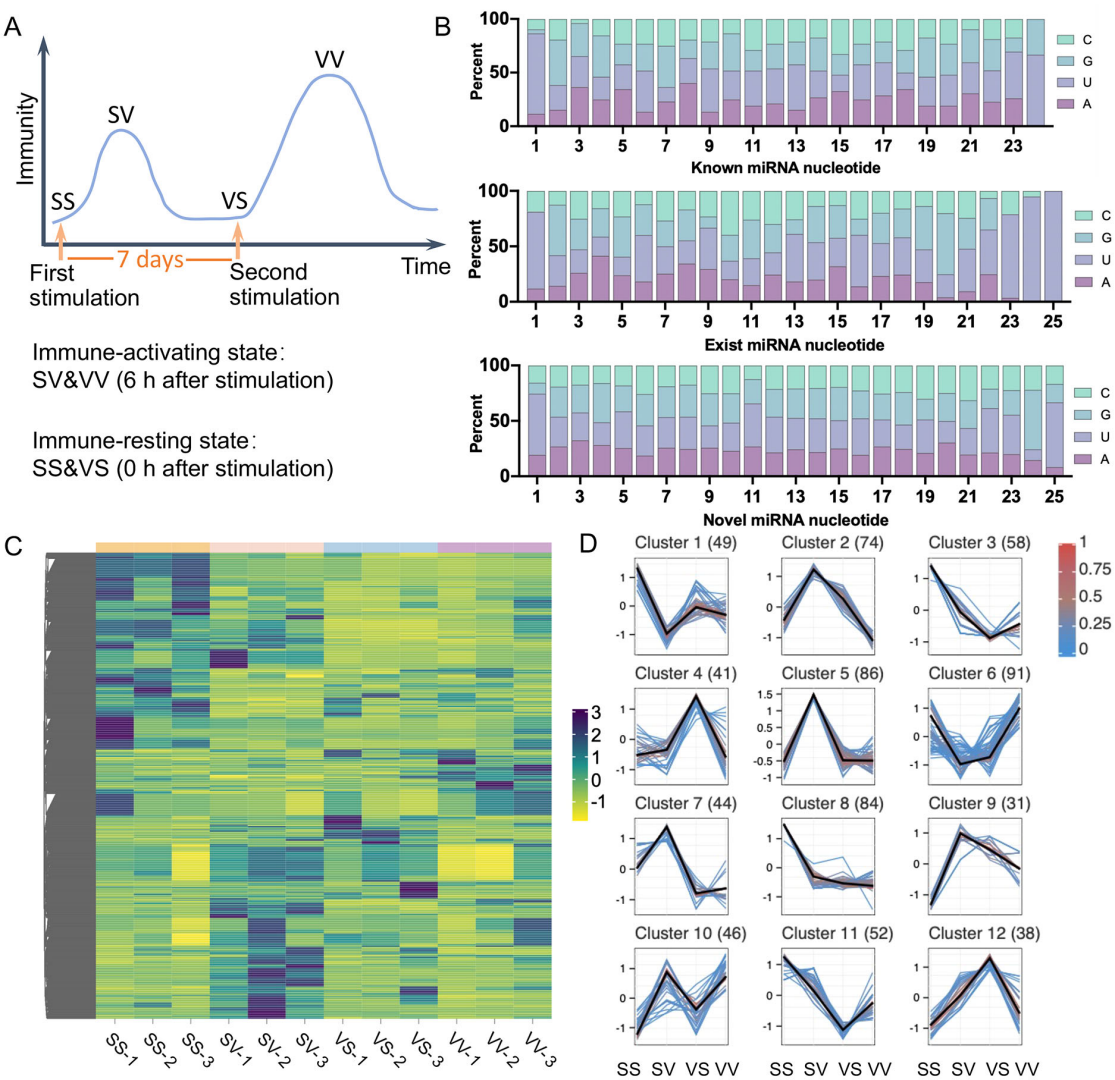
The miRNAs involved in the effect of *V.* sp*lendidus* pre-exposure and their expression heatmap among the four groups. **(A)** Model of immune priming in *C. gigas*, with SS, SV, VS and VV. The primary and secondary *V.* sp*lendidus* stimulations are separated by 7 days. **(B)** Nucleotide composition (C, G, U, A) across sequence positions (x-axis) for known, exist, and novel miRNAs, with y-axis representing nucleotide percentage. **(C)** Heatmap of the expression levels of identified miRNAs. The quantitative values corresponding to the color scale were normalized TPM values. **(D)** Identification of co-expressed miRNA clusters through Mfuzz analysis, where the central bold line denotes the cluster centroid, and surrounding lines illustrate individual miRNAs with fuzzy memberships.

To elucidate the potential molecular mechanisms for immune priming, which is characterized by elevated haemocyte counts, miRNA transcriptome sequencing of haemocytes was performed. Based on our previous study, 36 oysters were randomly assigned into four groups designated as the SS, SV, VS and VV groups (**Figure 1A**). In the SS and SV groups, the oysters received a first injection with 100 µL of sterile seawater, and a secondary injection with 100 µL of sterile seawater (SS) or a diluted suspension of live *V.* sp*lendidus* (4.0×10^8^ CFU mL^−1^) (SV) at 7 d after the first injection, respectively. In the VS and VV groups, the oysters were first stimulated with 100 µL of heat-killed *V.* sp*lendidus* (4.0×10^8^ CFU mL^−1^), and then treated with 100 µL of sterile seawater (VS) or 100 µL of live *V.* sp*lendidus* (VV) as the second stimulation at 7 d after the first injection, respectively. To account for the stress sensitivity of haemocytes, two times of injection with 100 µL of sterile seawater in SS group, and one injection with 100 µL of sterile seawater in SV or VS were designed. The use of inactivated bacteria, analogous to inactivated vaccines in vertebrates (8), enables safe delivery of the complete antigenic profile and induces an initial immune response. The time span of seven days was employed based on the previous reports in both vertebrates (21) and invertebrates (22, 23). For each group, haemocytes from three oysters were pooled at 6 h following the second *V.* sp*lendidus* injection. All samples were stored at -80 °C for subsequent RNA extraction and library preparation.

### 2.3 Identification of miRNAs

Following extraction with TRIzol reagent, total RNA was quantified and qualified by using a NanoDrop 2000 spectrophotometer (Thermo Fisher Scientific, America). Small RNA libraries were constructed and subsequently sequenced on the Illumina sequencing platform by Genedenovo Biotechnology (Guangzhou, China). The raw sequencing data were processed through a stringent filtering pipeline to obtain high-quality small RNA data. First, reads without 3’ adapters (3’ adapter-null) were removed. Subsequently, low-quality reads were filtered out by eliminating those containing more than one base with quality scores below 20 or any ambiguous ‘N’ bases, resulting in high-quality reads. Further filtration removed reads without insert fragments or with insert lengths shorter than 18 nucleotides. Additionally, reads containing poly A sequences (>70% of adenines bases) were excluded. The remaining high-confidence small RNA (sRNAs) sequences, termed clean sRNAs, were then used for downstream analyses. Sequence filtering was performed using in−house Perl scripts provided by Genedenovo Biotechnology (https://github.com/apanhui/miRNA_analysis_2). To remove known non-coding RNAs (rRNA, snRNA, snoRNA, tRNA), clean tags were filtered by alignment against the GenBank (Release 209.0) and Rfam (Release 211.0) databases using blastall (v2.2.25). Reads were mapped to the *C. gigas* genome (GCF_000297895.1) and aligned to MirGeneDB (https://mirgenedb.org) using Bowtie v1.1.2 with parameters -v 0 --best --strata -a for known miRNA identification, and novel miRNAs were predicted via hairpin structure analysis using MiRdeep2 (v2.0.0.7). Data obtained from the present study were deposited in the NCBI Sequence Read Archive under the accession number PRJNA1392860. edgeR (v3.12.1) was used to identify DE miRNAs, with a threshold of fold change ≥ 2 and *p* < 0.05.

### 2.4 Cluster analysis of miRNA

To identify clusters of miRNAs exhibiting similar expression pattern, the expression patterns of all miRNAs yielded by high-throughput sequencing were analyzed using Mfuzz (http://www.omicsmart.com).

### 2.5 Prediction of target genes and enrichment analysis of KEGG

The prediction of miRNA target genes was carried out using miRanda (v2.1.2), TargetScan (v7.0) and RNAhybrid (v3.3a) (24). The online platform Omicsmart (http://www.omicsmart.com) was used to perform KEGG enrichment analysis (Release 101) on the target genes of the screened DE miRNAs.

### 2.6 Gain-and loss-of-function assay of *Cgi*-miR-1175-P6/P7-y *in vivo*

*Cgi*-miR-1175-P6/P7-y was identified as a key miRNA for immune priming. To elucidate the function of *Cgi*-miR-1175-P6/P7-y in immune priming in oysters, gain-/loss-of-function assay were performed *in vivo* as previously described (25). A total of 45 oysters were selected and randomly assigned to five groups, designated as the sterile seawater group (SW), the mimics-treated group (*Cgi-*miR-1175-P6/P7-y mimics + VS), the mimics negative control group (mimics NC + VS), the inhibitor-treated group (*Cgi*-miR-1175-P6/P7-y inhibitor + VS) and the inhibitor negative control group (inhibitor NC + VS). The oysters in SW group received an injecton with 100 μL PBS, whereas other groups received injections of specific transfection complexes, each prepared by mixing 10 μL of mimic NC, mimic, inhibitor NC, or inhibitor with 10 μL of Lipo6000 and 80 μL of PBS. After 12 h, the SW group was stimulated with sterile seawater, while the other four groups were stimulated with *V.* sp*lendidus*. At 24 h post-stimulation, the hemolymph was collected using a 10 mL syringe and centrifuged at 700 × *g* for 12 minutes (min) at 4 °C. To assess haemocyte proliferation rates and the expression levels of *Cgi*-miR-1175-P6/P7-y and proliferation-related genes, haemocytes samples were collected from nine oysters per group and pooled into three biological replicates with three oysters per replicate. The sequences of *Cgi*-miR-1175-P6/P7-y mimics, mimics NC, *Cgi*-miR-1175-P6/P7-y inhibitor and inhibitor NC are synthesized by Genepharma (Suzhou, China) (**Table 1**).

**Table 1:** Sequences of the primers used in the present study.

Category	Primer name	Primer sequences (5’-3’)	
RNAs	*Cgi-*miR-1175-P6/P7-y mimics	CTTGGCACTGTCTGAGCGCAGGT CTGCGCTCAGACAGTGCCAAGTT
	*Cgi-*miR-1175-P6/P7-y mimics NC	TTCTCCGAACGTGTCACGTTT ACGTGACACGTTCGGAGAATT
	*Cgi-*miR-1175-P6/P7-y inhibitor	AGTTGGAGGAGTTGAATCTCAG	
	*Cgi-*miR-1175-P6/P7-y inhibitor NC	CAGTACTTTTGTGTAGTACAA	
Stem-loops reverse transcription	*Cgi-*miR-1175-P6/P7-y	GTCGTATCCAGTGCAGGGTCCGAGGTATTCGCACTGGATACGACAGTTGG
	U6	GACCGTGGCCCCTGGCGCTACAG
qRT-PCR	*Cgi-*miR-1993_3p	TATTATGCTGTTATTCACGAGA
	*Cgi-*miR-1990_3p	CGGGACTACGTCAACTACTTGC
	novel-m0115-5p	TGAGCATGACTGTGGACCC
	*Cgi-*miR-375-P3_3p	TTTGTTCAGTCGGCTCGCGTTA
	novel-m0275-3p	AATCTCAATGTCACAAGCAGCT
	*Cgi-*miR-1175-P6/7-y	CTGAGATTCAACTCCTCCAACT
	novel-m0095-3p	CCTTTAGCTGATTTTAACTA
	novel-m0147-5p	TTGCTTGGGTTTGAATGTTCAGA
	U6-F	GGAACGATACAGAGAAGATTAGC
	U6-R	TGGAACGCTTCACGAATTTGCG
	*Cgi-*miR-1175-P6/7-y-F	GCGCCTGAGATTCAACTCCT
	Stem-loops-U6 F	CCACGGTCCGCCCTCTATACAGT
	universal-R	GTGCAGGGTCCGAGGT
	*Cg*CDK6-F	GCGAAACCTCCACAGCCAATCA
	*Cg*CDK6-R	CGAGCGACAGTCCGACGTATCT
	*Cg*CDK14-F	AGTTATCGTTCTTGGTCTAGC
	*Cg*CDK14-R	CATGTATTGGCAGATGATAGT
	*Cg*SCARF2-F	TGTCTTCACCACCACCTGTCCT	
	*Cg*SCARF2-R	CAGCCAGCACCATACGTTCCTC	
	*Cg*Myc-A-F	TCAGGGCAAGTGGAAGTAGC
	*Cg*Myc-A-R	GGTGCACGTTCCTGCTTAAC
	*Cg*Runx-F	GTCTCCGCTGGAAACGATG
	*Cg*Runx-R	GTCACTTTGATGGCTTTCTGG
	*Cg*IL17-1-F	GCGAACGCCACAGTGTCAAA
	*Cg*IL17-1-R	GACGCTACGAGGAAATACGGAC
	*Cg*EF-F	AGTCACCAAGGCTGCACAGAAAG
	*Cg*EF-R	TCCGACGTATTTCTTTGCGATGT

### 2.7 The flow cytometry analysis of haemocyte proliferation

5-Ethynyl-2’-deoxyuridine (EdU) labeling was performed using Alexa Fluor 488 (Beyotime, China) (26). Haemocyte samples were fixed with 4% paraformaldehyde (Beyotime, China) for 15 min and then treated with a permeabilization solution (0.3% Triton X-100 in PBS, Beyotime, China) for 10 min. A portion of the sample was left unstained as a negative control. The remaining samples were stained with the Alexa Fluor 488 kit (C0071S, Beyotime, China) according to the manufacturer’s instructions. EdU-labeled circulating haemocytes were analyzed using a BD FACSAria II flow cytometer (BD Biosciences, USA), and data were processed with FlowJo software.

### 2.8 Quantitative real-time PCR analysis

According to the manufacturer’s instructions, the miRNA First Strand cDNA Synthesis (Sangon Biotech, China) was used to reverse transcribe miRNAs and the TransScript^®^ Uni All-in-One SuperMix (TransGene, China) was used to synthesize cDNA from 800 ng of total RNA. For reverse transcription in the *Cgi*-miR-1175-P6/P7-y functional experiments, the miRNA 1st strand cDNA synthesis kit (Accurate Biology, China) was employed. Quantitative real-time PCR (qRT-PCR) was performed with PerfectStart^®^ Green qPCR SuperMix (TransGene, China) to examine the miRNA expression levels of *Cgi*-miR-1175-P6/P7-y, *Cgi*-miR-1993_3p, *Cgi*-miR-1990_3p, novel-m0115-5p, *Cgi*-miR-375-P3_3p, novel-m0275-3p, novel-m0095-3p, novel-m0147-5p in four groups (SS, SV, VS and VV), as well as the expression levels of *Cgi*-miR-1175-P6/P7-y, cyclin-dependent kinase 6 (*Cg*CDK6), *Cg*CDK14, scavenger receptor class F member 2 (*Cg*SCARF2), *Cg*Myc-A, *Cg*Runx and Interleukin 17-1 (*Cg*IL17-1) in mimics- and inhibitor-treated groups, respectively. The amplification reaction was performed in a total volume of 10 μL containing 5 μL of 2×*PerfectStart*^®^ Green qPCR SuperMix (AQ602, TransGene, China), 2 μL of cDNA, 0.2 μL of 10 μM each primer, and 2.6 μL of ddH_2_O. qRT-PCR was performed on a QuantStudio™ 6 Flex instrument under the following cycling conditions: 94 °C for 30 s, followed by 40 cycles of 94 °C for 5 s and annealing for 32 s. The relative mRNA or miRNA expression was calculated using the 2^-ΔΔCt^ method, with Elongation factor (EF, NM_001305313) or U6 small nuclear 2 (U6, XR_004597400.1) as internal references. Detailed information on all the primers was listed in **Table 1**.

### 2.9 Statistical analysis

Statistical analysis of haemocyte proliferation rate and all qRT-PCR data was performed using SPSS 22.0. Differences between groups were assessed for significance using t-tests and One-Way ANOVA.

## 3 Results

### 3.1 The miRNA identified from the *C. gigas*

To investigate the *C. gigas* miRNA expression profiles in response to immune priming, the miRNA transcriptome sequencing of haemocytes was conducted. Twelve cDNA libraries were constructed using samples of SS 1-3, SV 1-3, VS 1-3, VV 1-3. A total of 115.13 M high-quality reads (SS = 27.89 M, SV = 30.14 M, VS = 28.24 M and VV = 28.87 M) were successfully yielded (**Table 2**). The four libraries yielded 8.02 to 11.35 million clean sRNAs, with unique mapping rates ranging from 70.33% to 82.75% (**Table 2**). After pooling and removing duplicates, a total of 694 miRNAs (51 known *C. gigas* miRNAs, 360 MirGeneDB miRNAs and 283 novel miRNAs) were identified as differentially expressed across all pairwise comparisons among the SS, SV, VS, and VV groups. A significant U-bias at the first nucleotide was observed across all known, existent, and novel miRNAs (**Figure 1B**), which is consistent with the previous study (27). The expression levels of the 694 miRNAs, quantified as transcripts per million (TPM), were clustered and visualized by heatmap (**Figure 1C**). Mfuzz clustering analysis further grouped these miRNAs into 12 distinct clusters based on their coordinated expression patterns across the four groups (**Figure 1D**).

**Table 2:** Processing quality statistics for small RNA sequencing data.

Sample	Clean reads	3’ adapter-null	High quality	Insert-null	Ploy A	Clean sRNAs
SS-1	11, 645, 116 (100%)	9, 050 (0.0783%)	11, 557, 439 (99.2471%)	332, 919 (2.8806%)	1, 453 (0.0126%)	9, 227, 872 (79.2424%)
SS-2	13, 515, 587 (100%)	15, 378 (0.1150%)	13, 373, 566 (98.9492%)	361, 129 (2.7003%)	1, 642 (0.0123%)	10, 635, 855 (78.6933%)
SS-3	9, 888, 943 (100%)	14, 194 (0.1441%)	9, 851, 052 (99.6168%)	231, 503 (2.3500%)	1, 024 (0.0104%)	8024689 (81.1481%)
SV-1	13, 497, 330 (100%)	17, 738 (0.1328%)	13, 355, 173 (98.9468%)	296, 653 (2.2213%)	2, 682 (0.0201%)	10, 470, 377 (77.5737%)
SV-2	13, 302, 042 (100%)	23, 022 (0.1744%)	13, 201, 112 (99.2412%)	329, 268 (2.4942%)	2, 187 (0.0166%)	10, 514, 824 (79.0467%)
SV-3	11, 061, 625 (100%)	28, 218 (0.2573%)	10, 968, 015 (99.1537%)	205, 288 (1.8717%)	1, 309 (0.0119%)	9, 154, 196 (82.7563%)
VS-1	10, 523, 864 (100%)	9, 288 (0.0889%)	10, 445, 086 (99.2514%)	309, 774 (2.9657%)	1, 423 (0.0136%)	8, 332, 560 (79.1778%)
VS-2	12, 927, 884 (100%)	16, 611 (0.1299%)	12, 790, 047 (98.9338%)	312, 306 (2.4418%)	1, 863 (0.0146%)	10, 213, 434 (79.0031%)
VS-3	13, 776, 284 (100%)	13, 862 (0.1017%)	13, 630, 255 (98.9400%)	584, 807 (4.2905%)	2, 590 (0.0190%)	9, 689, 939 (70.3378%)
VV-1	10, 488, 123 (100%)	24, 939 (0.2385%)	10, 454, 706 (99.6814%)	257, 063 (2.4588%)	1, 434 (0.0137%)	8, 411, 303 (80.1984%)
VV-2	14, 376, 462 (100%)	16, 139 (0.1125%)	14, 341, 647 (99.7578%)	424, 895 (2.9627%)	2, 884 (0.0201%)	11, 352, 191 (78.9637%)
VV-3	11, 887, 130 (100%)	15, 109 (0.1285%)	11, 758, 893 (98.9212%)	269, 213 (2.2894%)	2, 377 (0.0202%)	9, 102, 918 (76.5779%)

### 3.2 DE miRNAs that are involved in both primary and secondary immune responses

**Figure 2:**
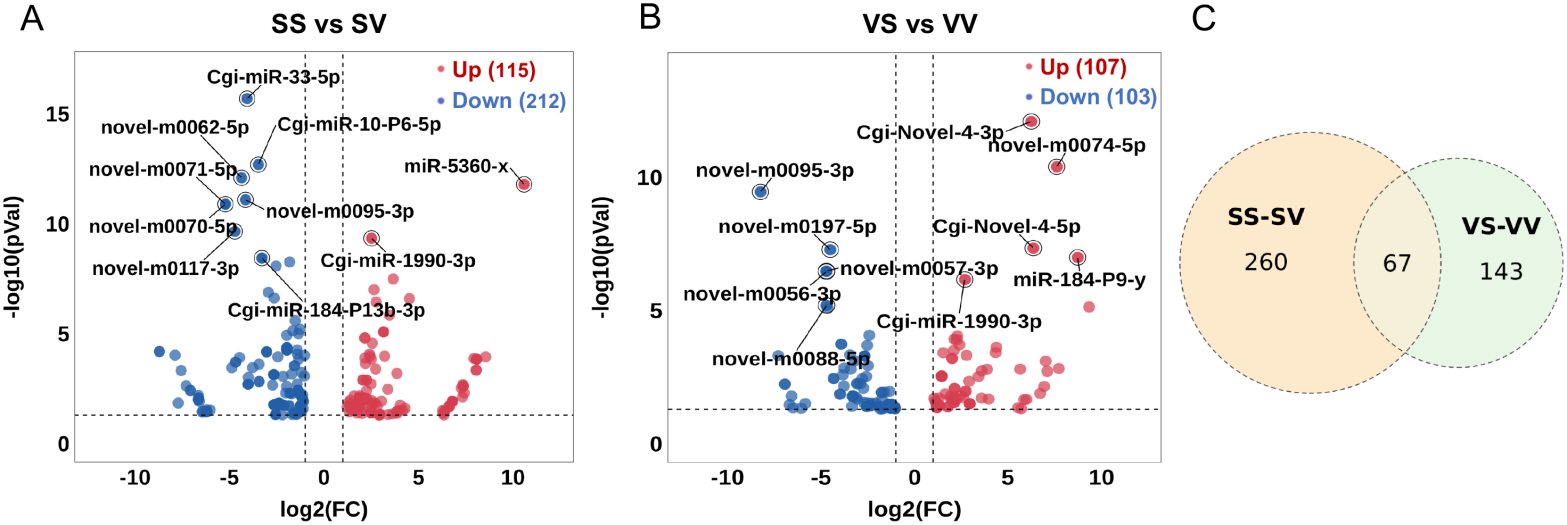
The common or specific DE miRNAs in the primary and secondary *V.* sp*lendidus* stimulations. **(A)** Volcano plot of DE miRNAs in the primary *V.* sp*lendidus* stimulations. The red dots represent up-regulated miRNAs, and blue dots represents down-regulated miRNAs. **(B)** Volcano plot of DE miRNAs in the secondary *V.* sp*lendidus* stimulations. The red dots represent up-regulated miRNAs, and blue dots represents down-regulated miRNAs. **(C)** Comparative overlap of DE miRNAs between the primary and secondary *V.* sp*lendidus* stimulations.

**Figure 3:**
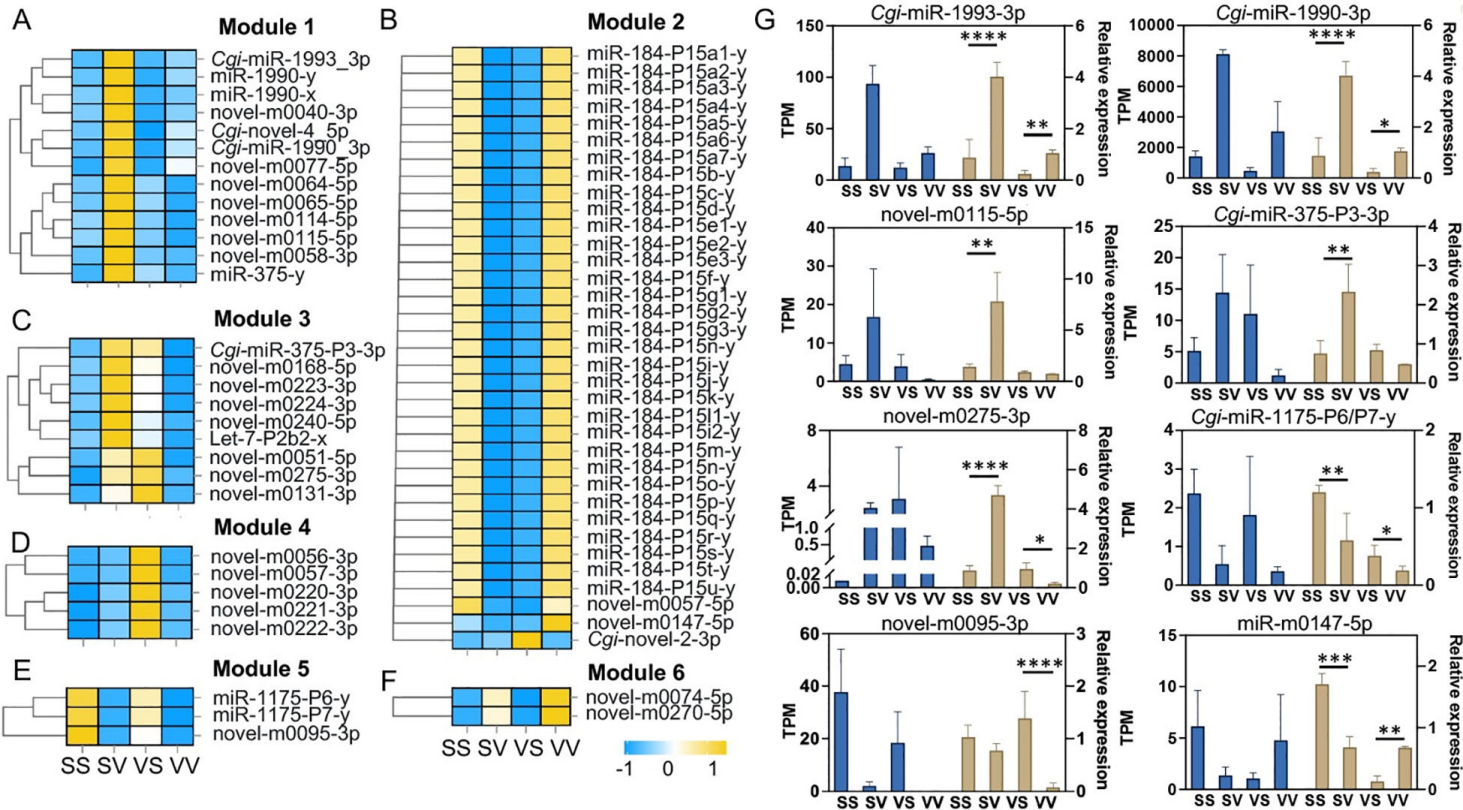
Heatmap and qRT-PCR validation of DE miRNAs differentially expressed in both the primary and secondary stimulations of *V.* sp*lendidus*. A total of 67 DE miRNAs exhibited distinct expression patterns and were divided into module 1 to 6 **(A-F)**. Columns represent different groups (SS, SV, VS and VV), and rows represents different of miRNAs. The quantitative values corresponding to the color scale were normalized TPM values. **(G)** The relative expression levels of eight miRNAs detected by qRT-PCR in the four groups. The asterisks indicate the significant differences compared with the control group (*p < 0.05; **p < 0.01; ***p < 0.001 and ****p < 0.0001).

The DE miRNAs were screened between SS-SV and VS-VV comparisons. The SS-SV comparison represents the response of non-primed oysters to a primary live *V.* sp*lendidus* stimulation and serves as the control for evaluating priming-specific effects in the VS-VV group. A total of 470 DE miRNAs were identified, with 115 up-regulated and 212 down-regulated miRNAs after the primary stimulation (SS-SV) (**Figure 2A**), and 107 up-regulated and 103 down-regulated miRNAs after the secondary stimulation (VS-VV) (**Figure 2B**, **Supplementary Table 1**). Notably, 67 miRNAs were found to be differentially expressed in both the primary and the secondary stimulations (**Figure 2C**). These 67 DE miRNAs exhibited distinct expression patterns and were divided into six modules (modules 1 to 6), containing 13, 35, 9, 5, 3 and 2 DE miRNAs, respectively (**Figures 3A–F**). After the primary stimulus, the DE miRNAs in module 2 and module 3 exhibited sustained low (**Figure 3B**) or high (**Figure 3C**) expression levels. Additionally, three DE miRNAs in module 5 (*Cgi*-miR-1175-P6-y, *Cgi*-miR-1175-P7-y, and novel-0095-3p) showed lower expression upon secondary stimulation compared to the primary response (**Figure 3E**), while novel-m0074-5p and novel-m0270-5p showed higher expression upon secondary stimulation (**Figure 3F**). The ‘-P6-y’ and ‘-P7-y’ suffixes in *Cgi-*miR-1175-P6-y and *Cgi-*miR-1175-P7-y reflect their relative positions within the gene clusters in the eutherian last common ancestor, while sharing identical mature sequences. This study collectively designates them as *Cgi*-miR-1175-P6/P7-y for simplicity. DE miRNAs in modules 5 and 6 have proposed to enhance immune responses. Meanwhile, DE miRNAs in modules 2 and 3, which maintained consistently low or high expression after the primary response, may contribute to immune memory formation or support energy provision for secondary stimulation. Eight of these 67 DE miRNAs were validated by qRT-PCR (**Figure 3G**), confirming the high reliability of the sequencing data.

### 3.3 KEGG enrichment analysis of the target genes of DE miRNAs

**Figure 4:**
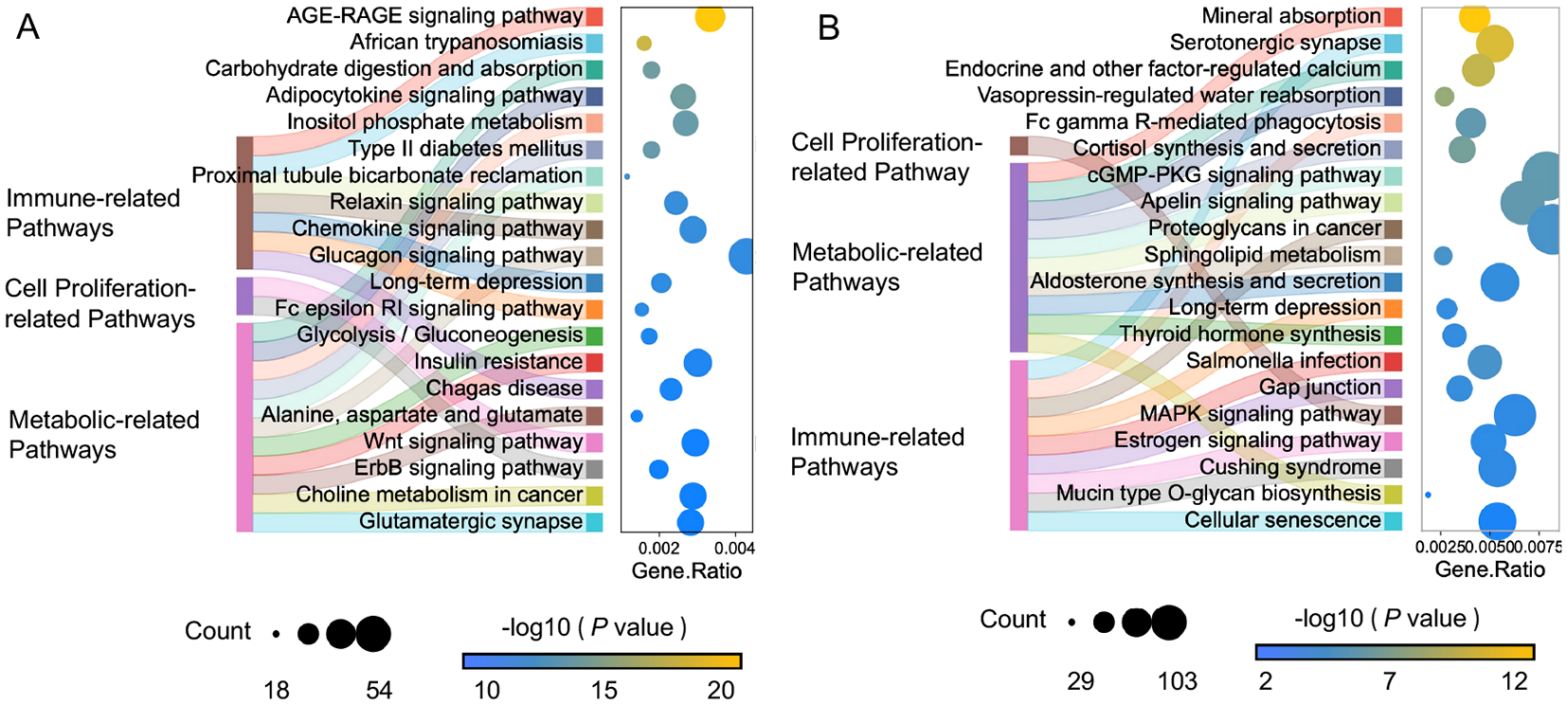
Sankey dot pathway enrichment analysis of the target genes of miRNAs in module 5 **(A)** and module 6 **(B)**. The x-axis shows Gene Ratio, and the y-axis shows enriched pathways (including categories of signaling pathways (left) and specific signaling pathways (right)). Dot size represents the count of target genes, and color intensity indicates -log_10_ (*P* value).

KEGG enrichment analysis was performed on the target genes of the putative immune enhancing DE miRNAs (module 5 and module 6) and the potential immune memory-related DE miRNAs (module 2 and module 3). The target genes of DE miRNAs in module 5 showed significant enrichment in cell proliferation-related signaling pathways (e.g., ErbB and wnt signaling) and metabolic reprogramming pathways (e.g., carbohydrate digestion and absorption, glycolysis/gluconeogenesis and insulin resistance). Module 5 was also enriched in immune-related signaling pathways, such as the AGE-RAGE, chemokine signaling pathway and Fc epsilon RI signaling pathways (**Figure 4A**). In contrast, module 6 miRNAs were primarily enriched in neuroendocrine- and neurotransmitter-related pathways, including serotonergic synapse, cortisol synthesis and secretion, thyroid hormone synthesis and estrogen signaling pathway (**Figure 4B**). Interestingly, the potential immune memory-related DE miRNAs in modules 2 and 3 were also associated with neuroendocrine (serotonergic synapse, cortisol synthesis and secretion, glutamatergic synapse and cushing syndrome) as well as intercellular communication(e.g. long-term depression and gap junction pathways) (**Supplementary Figure 1**). Together, these findings reveal a division of labor among miRNA modules, with module 5 driving immune activation and modules 2, 3, and 6 supporting systemic immune homeostasis.

### 3.4 Putative immune enhancing DE miRNAs targets multiple genes involved in cell proliferation, metabolism and immune response

**Figure 5:**
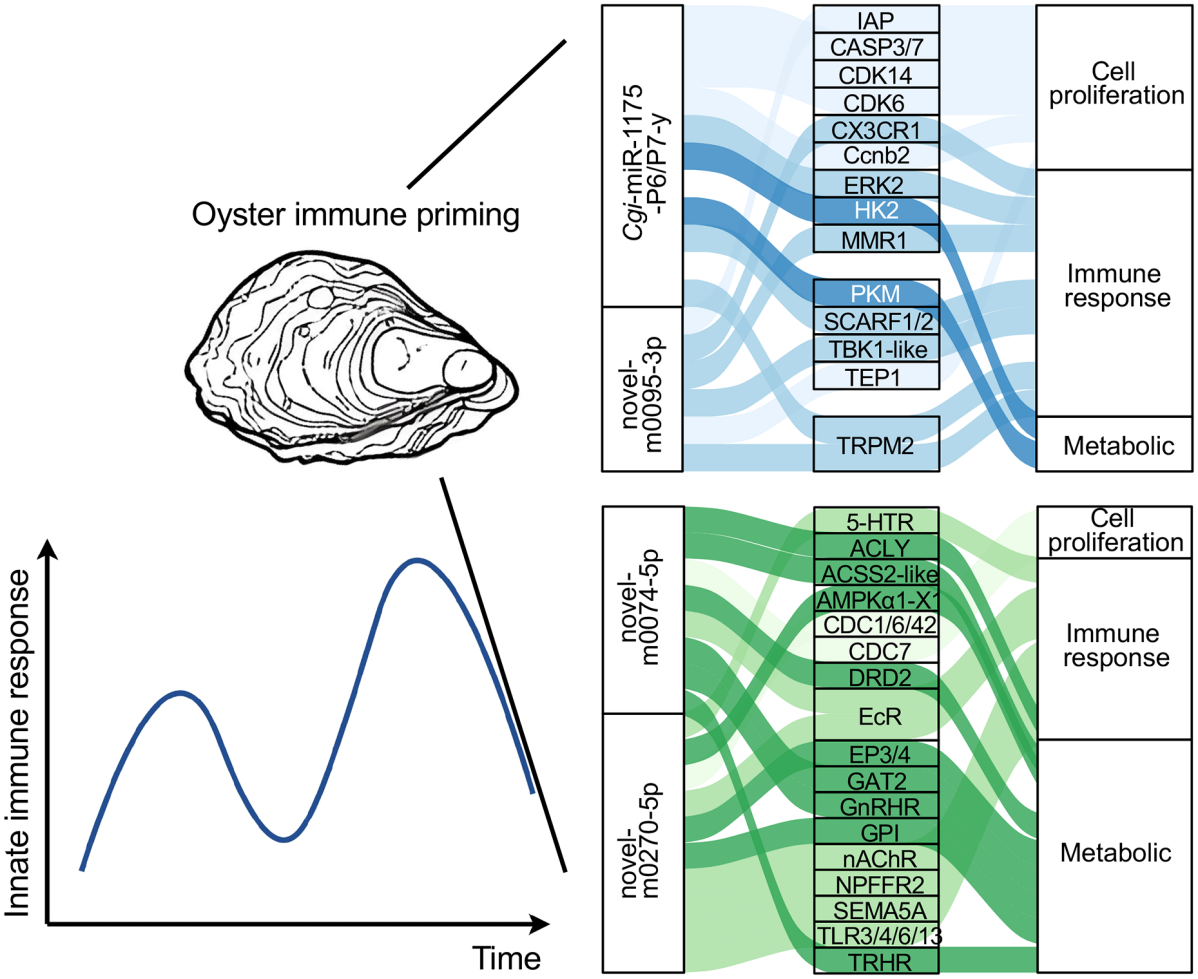
The target genes of five putative immune enhancing DE miRNAs in modules 5 and module 6. IAP, Inhibitor of apoptosis; CASP, Caspase; CDK, Cyclin-dependent kinase; CX3CR, chemokine receptor; Ccnb, G2/mitotic-specific cyclin; ERK, Extracellular signal-regulated kinase; HK, Hexokinase; MMR, Macrophage mannose receptor; PKM, Pyruvate Kinase PKM; SCARF, Scavenger receptor class F member; TBK1-like, Serine/threonine-protein kinase TBK like; TEP, Telomerase protein component; TRPM, Transient Receptor Potential Cation Channel Subfamily M Member; 5-HTR, 5-Hydroxytryptamine receptor; ACLY, ATP-citrate synthase; ACSS, Acetyl-coenzyme A synthetase; AMPKα1-X1, 5’-AMP-activated serine/threonine-protein kinase catalytic subunit alpha; CDC, Cell division control protein; DRD: D(2) dopamine receptor; EcR, Ecdysone receptor EP3/4, Prostaglandin E2 receptor EP3/4; GAT, GABA Transporter; GnRHR, Gonadotropin-releasing hormone receptor; GPI, Glucose-6-phosphate Isomerase; nAchR, Neuronal acetylcholine receptor; NPFFR, Neuropeptide FF receptor; SEMA, Semaphorin; TRHR, Thyrotropin-releasing hormone receptor.

Based on the KEGG enrichment results, target genes of miRNAs in modules 5 and 6 were further investigated. The target genes of five putative immune enhancing DE miRNAs in modules 5 and module 6 were further screened, respectively. The expression level of *Cgi*-miR-1175-P6/P7-y, which was significantly decreased upon secondary stimulation, may enhance immune responses or promote cell proliferation by releasing post-transcriptional repression of multiple pattern recognition receptors (PRRs), cell cycle-related genes and glycolytic-related genes, including SCARF1/2, CDK6/14, hexokinase 2 (HK2) and pyruvate kinase PKM (PKM). Meanwhile, novel-miR-0095-3p may target mannose receptor and cell survival-related genes, such as macrophage mannose receptor 1 (MMR1), C-type mannose receptor 2 (MRC2), inhibitor of apoptosis (IAP) and telomerase protein component (TEP) (**Figure 5**). Additionally, target gene analysis revealed that the novel-m0074-5p and novel-m0270-5p modulate various neuroendocrine receptors, such as 5-Hydroxytryptamine receptor (5-HTR), ecdysone receptor (EcR), neuronal acetylcholine receptor (nAChR), D (2) dopamine receptor (DRD2), neuropeptide FF receptor 2(NPFFR2), gonadotropin-releasing hormone receptor (GnRHR), thyrotropin-releasing hormone receptor (TRHR), prostaglandin E2 receptor EP3/4 (EP3/4), as well as neurotransmitter transporters (e.g., GABA transporters, GAT), thereby maintaining homeostasis of the immune micro-environment.

### 3.5 *Cgi-*miR-1175-P6/P7-y regulates cell proliferation by mediating cyclin-dependent kinase

**Figure 6:**
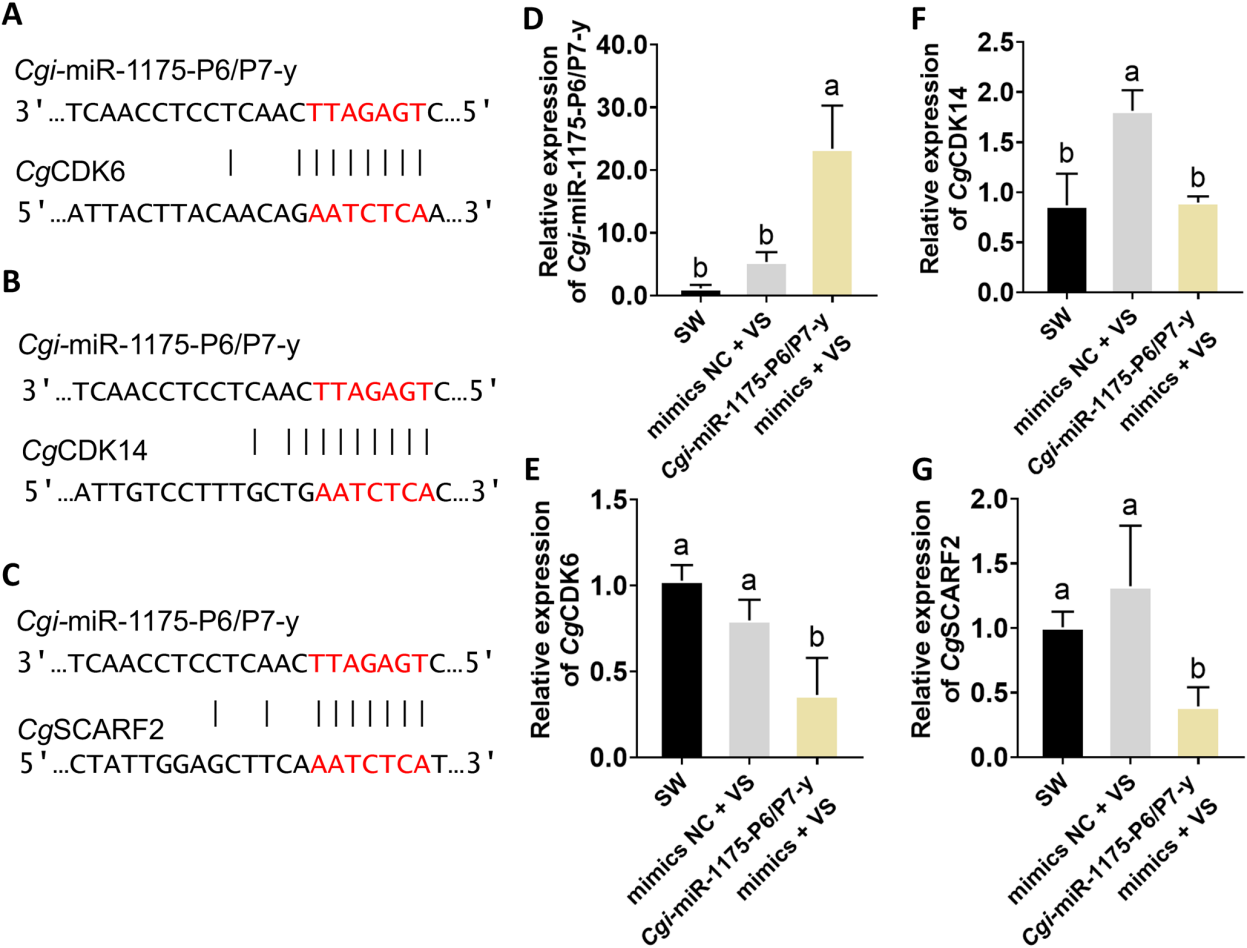
Binding site prediction and the expression levels of between *Cgi*-miR-1175-P6/P7-y and target genes. **(A-C)** Binding sites for *Cgi*-miR-1175-P6/P7-y were predicted within *Cg*CDK6, *Cg*CDK14 and *Cg*SCRAF2. The seed region of the miRNA (complementary sequence, red) is highlighted. The expression levels of *Cgi-*miR-1175-P6/P7-y **(D)**, *Cg*CDK6 **(E)** and *Cg*CDK14 **(F)** and *Cg*SCARF2 **(G)** in gain-of-function assay. Vertical bars represent mean ± S.D. (N = 6). Significant differences (*p* < 0.05) among groups are denoted by distinct lowercase letters (a–b).

Since *Cgi*-miR-1175-P6/P7-y in module 5 was predicted to target multiple cell proliferation-related genes, its biological function in oysters during immune priming was further validated. *Cg*CDK6, *Cg*CDK14 and *Cg*SCARF2 were identified as the putative target gene of *Cgi*-miR-1175-P6/P7-y. Binding site between *Cgi*-miR-1175-P6/P7-y and *Cg*CDK6, *Cg*CDK14 and *Cg*SCARF2 was shown in **Figures 6A–C**.

**Figure 7:**
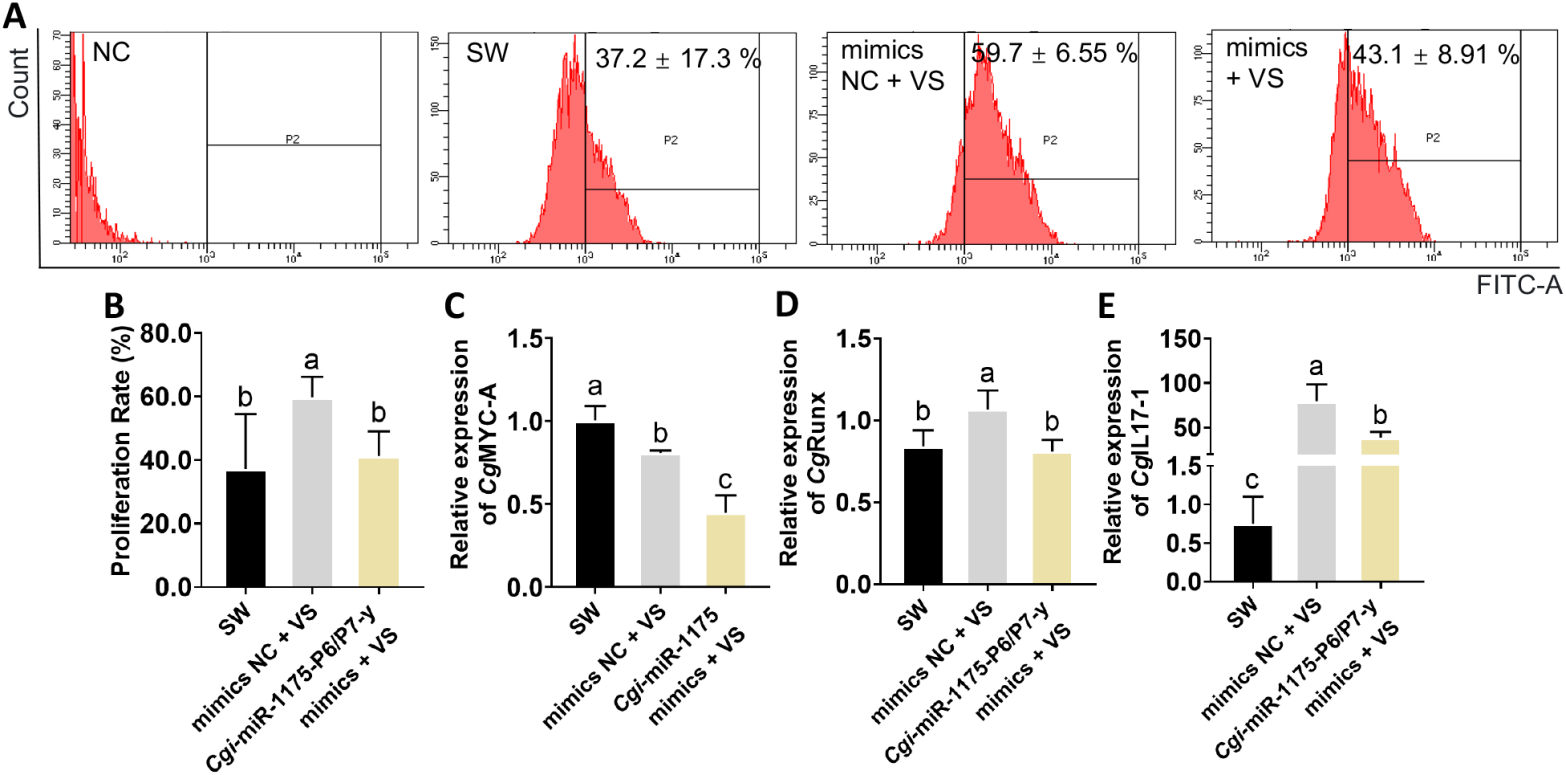
*In vivo* injection of *Cgi-*miR-1175-P6/P7-y mimics inhibited haemocyte proliferation and mRNA expression levels of related genes after *V.* sp*lendidus* stimulation. **(A)** The EdU^+^ haemocytes in oysters. The FITC threshold for EDU-labeled proliferating cells identification was established using unstained negative controls. The percentage of EdU^+^ haemocytes in oysters after the stimulation with sterilized seawater, mimics NC group and *Cgi-*miR-1175-P6/P7-y mimics-treated group. Percentages reflect the proportion of EdU^+^ haemocytes, while dot plots display FITC-A fluorescence intensity (red dots: EdU^+^ proliferating cells, unlabeled: non-proliferating cells). **(B)** The statistical results of proliferation rates. The mRNA expression levels of *Cg*Myc-A **(C)**, *Cg*Runx **(D)** and *Cg*IL17-1 **(E)** in gain-of-function assay. Vertical bars represent mean ± S.D. (N = 6). Significant differences (*p* < 0.05) among groups are denoted by distinct lowercase letters (a–c).

**Figure 8:**
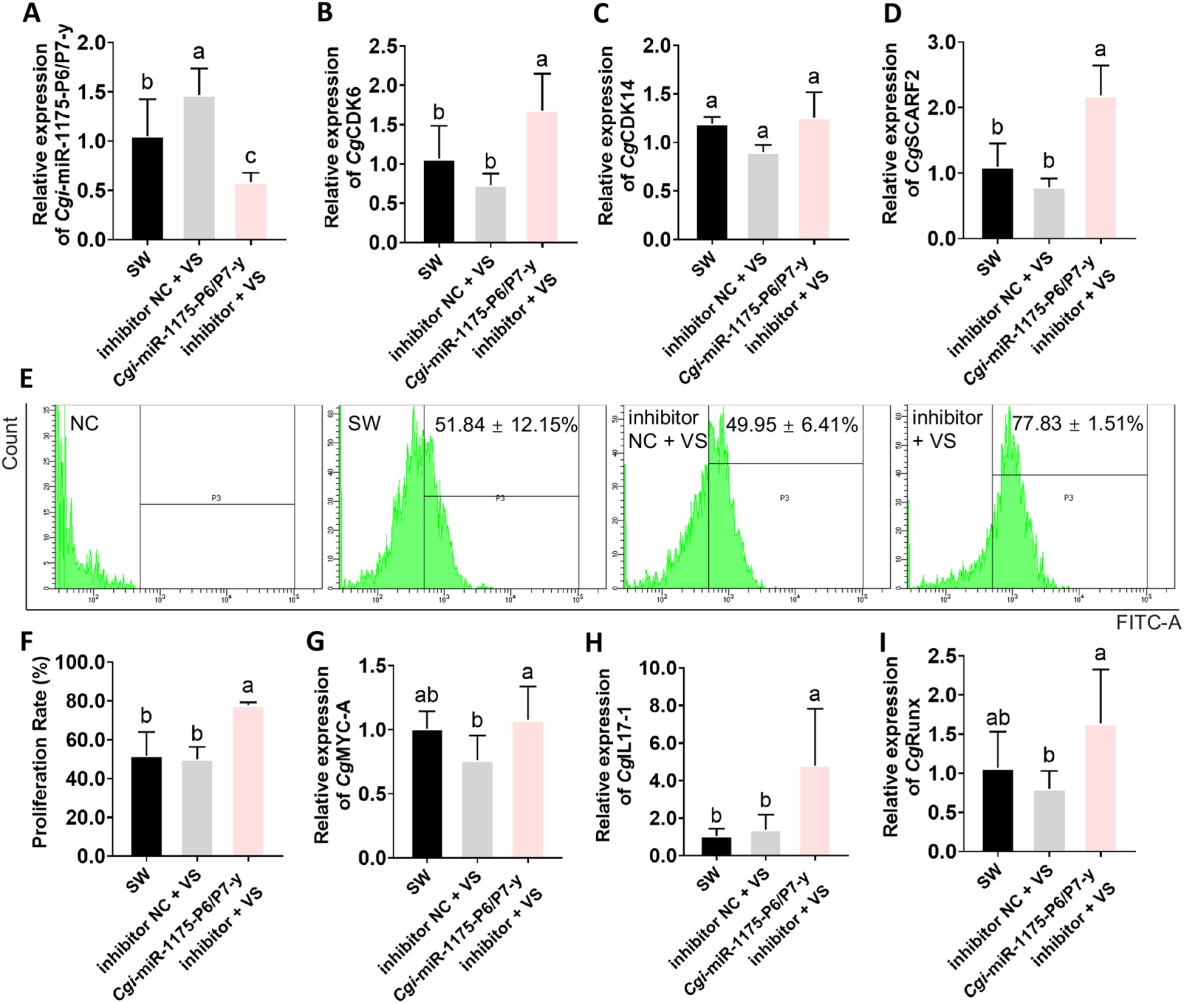
*In vivo* injection of *Cgi-*miR-1175-P6/P7-y inhibitor promoted haemocyte proliferation and mRNA expression levels of related genes after *V.* sp*lendidus* stimulation. The expression levels of *Cgi-*miR-1175-P6/P7-y **(A)**, *Cg*CDK6 **(B)***Cg*CDK14 **(C)** and *Cg*SCARF2 **(D)** in loss-of-function assay. **(E)** The EdU^+^ haemocytes in oysters. The FITC threshold for EdU-labeled proliferating cells identification was established using unstained negative controls. The percentage of EdU^+^ haemocytes in oysters after the stimulation with sterilized seawater, inhibitor NC group and *Cgi-*miR-1175-P6/P7-y inhibitor-treated group. Percentages reflect the proportion of EdU^+^ haemocytes, while dot plots display FITC-A fluorescence intensity (green dots: EdU^+^ proliferating cells, unlabeled: non-proliferating cells). **(F)** The statistical results of proliferation rates. The mRNA expression levels of *Cg*Myc-A **(G)**, *Cg*IL17-1 **(H)** and *Cg*Runx **(I)** in loss-of-function assay. Vertical bars represent mean ± S.D. (N = 6). Significant differences (*p* < 0.05) among groups are denoted by distinct lowercase letters (a–c).

Gain/loss-of-function assay were performed to elucidate the regulatory role of *Cgi*-miR-1175-P6/P7-y in oyster haemocyte proliferation. The mimics-treated group exhibited a 4.37-fold up-regulation of *Cgi*-miR-1175-P6/P7-y relative to the NC group (*p* < 0.0001, **Figure 6D**). Furthermore, the mRNA expression levels of *Cg*CDK6, *Cg*CDK14 and *Cg*SCARF2 were lower than that of in the mimics NC group (**Figures 6E-G**). The FITC threshold to identify EdU-positive cells was determined using unstained cell controls (**Figure 7A**). Following *V.* sp*lendidus* stimulation, treatment with *Cgi*-miR-1175-P6/P7-y mimics reduced the percentage of EdU^+^ haemocytes to 0.72-fold that of the mimics NC group (**Figure 7B**) and decreased the mRNA expression levels of *Cg*Myc-A, *Cg*Runx, and *Cg*IL17–1 to 0.59, 0.75, and 0.48-fold, respectively (*p* < 0.05, **Figures 7C–E**). Conversely, the inhibitor-treated oysters exhibited a 0.60-fold down-regulation of *Cgi*-miR-1175-P6/P7-y relative to the inhibitor NC group (*p* < 0.0001, **Figure 8A**). Furthermore, the mRNA expression levels of *Cg*CDK6, *Cg*CDK14 and *Cg*SCARF2 in the inhibitor-treated group were higher than those in the inhibitor NC group (**Figures 8B–D**). After treatment with *Cgi*-miR-1175-P6/P7-y inhibitor the percentage of EdU^+^ haemocytes was increased to 1.56-fold that of the inhibitor NC group following *V.* sp*lendidus* stimulation (**Figures 8E, F**), and the mRNA expression levels of *Cg*Myc-A, *Cg*IL17-1, and *Cg*Runx also increased to 1.41, 2.03, and 3.48-fold, respectively (**Figures 8G–I**, *p* < 0.05). Collectively, these reciprocal results demonstrate that *Cgi*-miR-1175-P6/P7-y acts as a key negative regulator of haemocyte proliferation in oysters.

## 4 Discussion

As molecular rheostats, miRNAs precisely orchestrate immune responses by finely regulating gene expression. Recent studies highlight their growing importance in mediating epigenetic modifications and metabolic reprogramming in immune memory (14, 17). Therefore, the exploration of miRNA regulating immune-activated genes could provide a crucial foundation for elucidating the mechanisms behind enhanced immune response in invertebrates. In the present study, the expression profiles of miRNAs and their biological functions in *C. gigas* after the primary and secondary *V.splendidus* stimulations were investigated.

miRNA transcriptome sequencing identified 411 known and 283 novel miRNAs in *C. gigas*. The number of known miRNAs and novel miRNAs detected in this study was much higher than that of *V.splendidus*-infected *C. gigas* (28), low salinity-stressed *C. gigas* and *C. hongkongensis* (29), WSSV-infected *Fenneropenaeus penicillatus* (30) and heat-stressed *Apostichopus japonicus* (31). One of the potential reasons for the discrepancy is the updated MirGeneDB 3.0 (more than 21, 000 microRNA gene entries) employed in this study (32), in contrast to the miRBase versions (19.0-22.0) used in previous studies. miRNA transcriptome sequencing revealed numerous DE hemocyte miRNAs after the primary and secondary stimulations.

Within the regulatory network of immune priming, miRNAs that exhibit a sustained response after the primary stimulation or more significant changes upon the secondary stimulation are crucial. A total of 67 miRNAs that were differentially expressed in both the primary and secondary stimulations (**Figure 3**) and were categorized into 6 modules based on their expression patterns, suggesting their indispensable roles in the immune response. Notably, neuroendocrine (serotonergic synapse, cortisol synthesis and secretion, glutamatergic synapse and cushing syndrome) and intercellular communication (long-term depression and gap junction) pathways were simultaneously enriched among the potential immune memory-related DE miRNAs in modules 2 and 3 (**Figure 3**). The activated neuroendocrine system regulates immune effector production via neurotransmitter-receptor binding (33). For instance, serotonin binds to the receptor *Cg*5-HTR-1 to regulate the synthesis of tumor necrosis factor (*Cg*TNF) and the apoptosis of hemocyte (34). Additionally, hormones such as cortisol regulate immune status in *Atlantic salmon* through influencing the dynamic expression of immune-related genes during parasitic infection (35). Notably, long-term depression and gap junctions contribute significantly to the intercellular communication network between neuroendocrine and immune systems under stress conditions (36, 37). The results indicated that DE miRNAs in modules 2 and 3, with opposing expression trends, coordinate cytokine production, energy allocation, and intercellular communication. This synergy maintains homeostasis and primes the oyster into a “pre-activated” state of alertness for secondary stimulations. Potential immune memory-related DE miRNAs in module 5 were enriched in metabolic-related pathways (glucagon signaling, insulin resistance and glycolysis/gluconeogenesis) and cell proliferation-related pathways (wnt and ErbB signaling pathways) (**Figure 4**). In mammals, tissue hypoxia induces insulin resistance by triggering a metabolic switch to aerobic glycolysis (38). It could be inferred that in invertebrates, Insulin resistance and glycolysis/gluconeogenesis metabolism regulated by DE miRNAs in Module 5 also play indispensable roles in immune activation. Additionally, previous study established that the wnt and ErbB signaling pathways collectively regulate cell proliferation by inducing c-Myc expression (39) and modulating cell-cycle arrest (40), respectively. These results indicated that the DE miRNAs in module 5 orchestrated immune response by coordinately regulating metabolic and cell proliferation processes. DE miRNAs in module 6 were enriched in neuroendocrine- and neurotransmitter-related pathways, including serotonergic synapse, cortisol synthesis and secretion, thyroid hormone synthesis and estrogen signaling pathway, etc. In oysters, the enzymes involved in cortisol metabolism have been identified as regulators of glycogen metabolism (41). The results collectively supported that miRNAs in module 5 may enhance immune responses by mediating cell proliferation and metabolic processes, whereas miRNAs in modules 2, 3, and 6 maintain immunological homeostasis through systemic regulation of the neuroendocrine system, modulating cytokine production and energy allocation.

The primary objective of this study was to investigate the potential miRNA-target gene network that supports enhanced immune response. As shown in **Figure 5**, *Cgi*-miR-1175-P6/P7-y were predicted to target multiple PRRs (SCARF1 and SCARF2), cell cycle-related genes (CDK6 and CDK14) and glycolytic-related genes (HK2 and PK), whereas novel-m0095-3p targeted mannose receptor (MMR1) and cell survival-related genes (TEP and IAP). Scavenger receptors has known to act as versatile pattern recognition molecules by initiating phagocytosis and signal transduction, thereby influencing downstream cellular responses (42). Upon receiving these signals, CDKs function as critical regulators of cell proliferation. TEP and IAP maintain a balance between cell proliferation and cell death (43, 44). Our results collectively suggest that novel-m0095-3p and *Cgi*-miR-1175-P6/P7-y may influence haemocyte regeneration by modulating cell proliferation or cell death. Additionally, novel−m0074−5p and novel−m0270−5p were predicted to target a diverse set of neuroendocrine receptors (5-HTR, EcR, nAChR, DRD2, NPFFR2, GnRHR, TRHR and EP3/4) and GAT, respectively. Previous studies in oysters have demonstrated the existence of neuroendocrine regulation of immunity. Specifically, *Cg*mAChR-1, *Cg*EP4, *Cg*5-HTR, and the *Cg*GABA have been demonstrated to mediate immune responses by modulating the expression levels of inflammatory cytokines (34, 45, 46). In summary, the results suggested that *Cgi*-miR-1175-P6/P7-y and novel-m0095-3p may enhance circulating haemocyte regeneration by targeting cell proliferation-related genes, whereas novel-m0074-5p and novel-m0270-5p maintain immune homeostasis by regulating cytokines through target neuroendocrine factors in oyster immune priming.

Immune response mediated by cell proliferation has been demonstrated in various primed invertebrates. Primed oysters show a significant increase in cell numbers within 6 hours post-secondary challenge (12), while primed *Biomphalaria snails* (24 h) (10) and shrimp (3 d) (6) exhibit similar responses at later time points. This variation may depend on the specific defense mechanisms of organism. It has showed that *Cgi*-miR-1175-P6/P7-y negatively regulated cell proliferation. Through *in vivo* injections of *Cgi*-miR-1175-P6/P7-y mimics, both the rate of EdU^+^ haemocytes (**Figures 7A, B**) and the mRNA expression levels of target genes (*Cg*CDK6, *Cg*CDK14 and *Cg*SCARF2) (**Figures 6E–G**) and proliferation-related genes (*Cg*Myc-A, *Cg*Runx and *Cg*IL17-1) (**Figures 7C–E**) were reduced following *V.* sp*lendidus* stimulation. It has been demonstrated that *Cgi*-miR-1175-P6/P7-y negatively regulates haemocyte proliferation in oysters. CDKs are essential for driving cell proliferation, as they coordinate the critical events of DNA replication and division throughout the cell cycle. Extensive studies have established that the involvement of cyclin-dependent kinases in cell proliferation regulation can be targeted and modulated by specific miRNAs. For example, CDK14 is repressed by miR-1-3p to inhibit osteosarcoma proliferation (47), and CDK6 is targeted by miR-34a to induce cell cycle arrest (48). It has been documented that CDK14 phosphorylates LRP5/6 to activate Wnt/β−catenin−mediated transcription of MYC (49), whereas in papillary thyroid cancer, the suppression of MYC has been found to downregulate its downstream targets CDK4, CDK6, and p21 (50). These results imply that MYC may serve as a central signaling hub connecting CDK14 and CDK6 in the regulation of cell proliferation. As above, decreased expression levels of *Cgi*-miR-1175-P6/P7-y after *V.* sp*lendidus* stimulation may lead to haemocyte proliferation by alleviating its suppression on CDKs (*Cg*CDK6 and *Cg*CDK14), which may be further modulated by MYC through potential cross−talk (**Figures 7C**, **8G**).

## 5 Conclusions

A comprehensive investigation of miRNA expression profiles of oyster during immune priming was performed. There were 115 and 107 miRNAs exhibited up-regulation after the primary and secondary stimulation, while 212 and 103 miRNAs showed down-regulation. Among these DE miRNAs, *Cgi*-miR-1175-P6/P7-y and novel-m0095-3p may enhance immune responses by mediating cell proliferation and metabolic processes, whereas DE miRNAs in modules 2, 3 and 6 maintain immunological homeostasis through systemic regulation of the neuroendocrine system, modulating cytokine production and energy allocation. Functional experiments demonstrated that haemocyte proliferation was negatively regulated by *Cgi*-miR-1175-P6/P7-y via targeting of *Cg*CDK6, *Cg*CDK14 and *Cg*SCARF2. The miRNAs identified in this study provide a valuable resource for advancing the understanding in miRNA function in immune priming of oyster.

## Data Availability

The datasets presented in this study can be found in online repositories. The names of the repository/repositories and accession number(s) can be found in the article/**Supplementary Material**.

## Supplementary material

Supplementary Figure 1 KEGG pathway enrichment analysis of target genes of DEmiRNAs in module 2 **(A)** and module 3 **(B)**. The top 20 most significantly enriched pathways were shown. The x-axis represents Gene Ratio, and the y-axis lists enriched pathways. Bubble size respresents the number of target genes in the pathway, and color intensity reflects -log_10_(Q-value).
