# Supplementary figures and images for: The involvement of miRNAs in the enhanced immune response of oysters via haemocyte-mediated immune priming
Source: Front Immunol. 2026 Mar 4;17:1753252. doi: 10.3389/fimmu.2026.1753252 (PMC12995623; doi:10.3389/fimmu.2026.1753252)

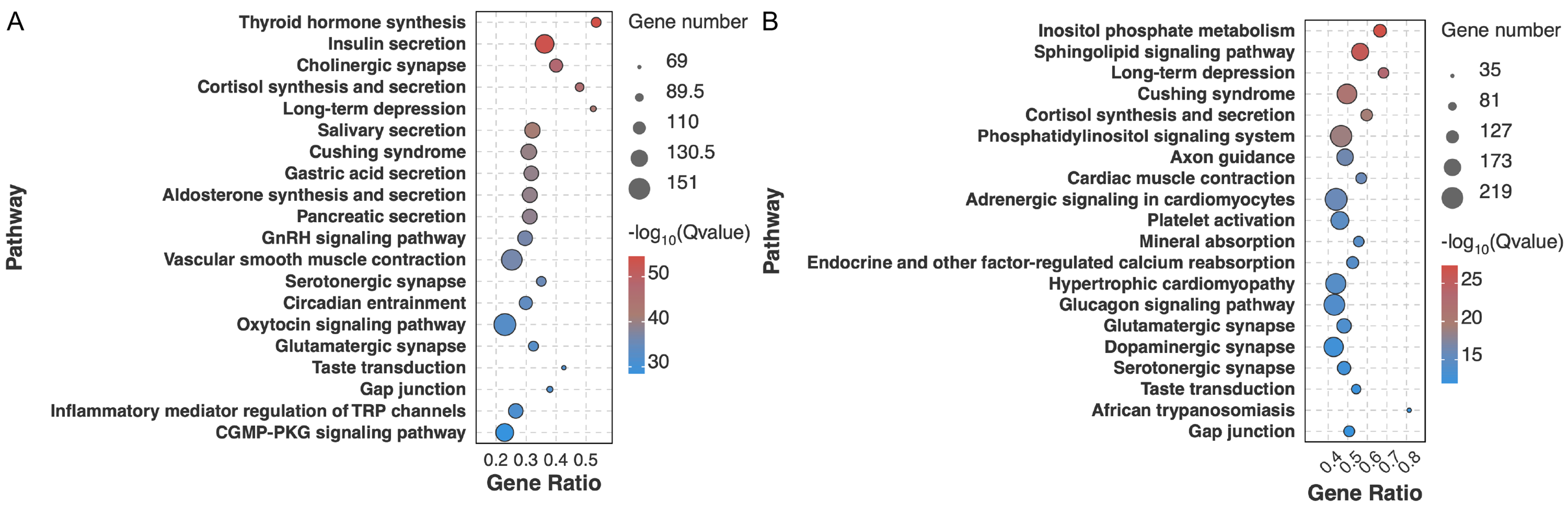

Supplement: Supplementary Figure 1 — KEGG pathway enrichment analysis of target genes of DEmiRNAs in module 2 (A) and module 3 (B). The top 20 most significantly enriched pathways were shown. The x-axis represents Gene Ratio, and the y-axis lists enriched pathways. Bubble size respresents the number of target genes in the pathway, and color intensity reflects -log10(Q-value). [file Image1.tif]
